# Effects of Different Concurrent Resistance and Aerobic Training Frequencies on Muscle Power and Muscle Quality in Trained Elderly Men: A Randomized Clinical Trial

**DOI:** 10.14336/AD.2016.0504

**Published:** 2016-12-01

**Authors:** Rodrigo Ferrari, Sandra C. Fuchs, Luiz Fernando Martins Kruel, Eduardo Lusa Cadore, Cristine Lima Alberton, Ronei Silveira Pinto, Régis Radaelli, Maira Schoenell, Mikel Izquierdo, Hirofumi Tanaka, Daniel Umpierre

**Affiliations:** ^1^Postgraduate Studies Program in Cardiology, School of Medicine, Universidade Federal do Rio Grande do Sul, Porto Alegre, RS, Brazil; ^2^Exercise Pathophysiology Research Laboratory, Cardiovascular Division, Hospital de Clínicas de Porto Alegre, Porto Alegre, RS, Brazil; ^3^Exercise Laboratory Research, Physical Education School, Universidade Federal do Rio Grande do Sul, Porto Alegre, RS, Brazil; ^4^Department of Health Sciences, Public University of Navarre, Navarre, Spain; ^5^Cardiovascular Aging Research Laboratory, University of Texas at Austin, TX 78712, USA

**Keywords:** Exercise, combined training, resistance training, aerobic training, aging, functional outcomes

## Abstract

Muscle power is a strong predictor of functional status in the elderly population and is required to perform different daily activities. To compare the effects of different weekly training frequencies on muscle power and muscle quality induced by concurrent training (resistance + aerobic) in previously trained elderly men. Twenty-four trained elderly men (65 ± 4 years), previously engaged in a regular concurrent training program, three times per week, for the previous five months, were randomly allocated to concurrent training programs in which training was performed either twice a week (2·week^-1^, n = 12) or three times per week (3·week^-1^, n = 12). The groups trained with an identical exercise intensity and volume per session for 10 weeks. Before and after the exercise training, we examined muscle power, as estimated by countermovement jump height; knee extensor isokinetic peak torque at 60 and 180^o.^s^-1^; and muscle quality, a quotient between the one-repetition maximum of the knee extensors and the sum of quadriceps femoris muscle thickness determined by ultrasonography. Additionally, as secondary outcomes, blood pressure and reactive hyperemia were evaluated. Two-way ANOVA with repeated measures were used and statistical significance was set at α = 0.05. Muscular power (2·week^-1^: 7%, and 3·week^-1^: 10%) and muscle quality (2·week^-1^: 15%, and 3·week^-1^: 8%) improved with the concurrent exercise training (*p* < 0.001) but with no differences between groups. The isokinetic peak torque at 60 (2·week^-1^: 4%, and 3·week^-1^: 2%) and 180^o.^s^-1^ (2·week^-1^: 7%, and 3·week^-1^: 1%) increased in both groups (*p* = 0.036 and *p*=0.014, respectively). There were no changes in blood pressure or reactive hyperemia with the concurrent training. Concurrent training performed twice a week promotes similar adaptations in muscular power and muscle quality when compared with the same program performed three times per week in previously trained elderly men.

The marked reductions in cardiovascular and neuromuscular functions that occur with advancing age[1] can lead to a loss of mobility[2] as well as higher risks of mortality[3] in elderly populations. Resistance training has been recommended as the first-line strategy to improve age-related loss of skeletal muscle mass, strength, and power[4, 5]. It has been suggested that muscular power is a more discriminant predictor of functional performance in older adults than muscle strength[6]. Similarly, muscle quality (i.e., force per unit of muscle mass) seems to be other important variable associated with functional capacity in elderly [7]. Considering that these variables are strong predictors of functional status in the elderly population and are required to perform different daily activities, such as walking, climbing stairs, or simply standing from a seated position[8], exercise interventions that enhance muscular power and muscle quality along with aerobic capacity should be highlighted in older adults[9]. In addition, habitual aerobic exercise can minimize several adverse physiological changes associated with aging, such as low aerobic capacity, hypertension, arterial stiffening and endothelial dysfunction [10-13].

In the scheme of exercise training prescription, training stimuli are gradually augmented by increasing the duration and/or frequency as the trainee becomes fitter in the exercise training programs. Interestingly, aerobic or resistance training performed exclusively (twice a week) and concurrent training performed with resistance exercise on one day and aerobic exercise on the other day produced similar increases in cardiovascular and neuromuscular parameters in previously untrained elderly men[14]. However, studies evaluating the effects of different frequencies of concurrent training on neuromuscular and cardiovascular functions are limited [15, 16], especially in previously trained older adults[16]. Moreover, there are no data comparing different weekly frequencies of concurrent training on power and muscle quality adaptations in the elderly.

Most of the available information comes from exercise training intervention studies targeted for previously sedentary populations[17]. Currently, no information is available that indicates whether different training frequencies would affect training adaptation among older adults who have been regularly exercising. Therefore, the purpose of the present study was to compare the effects of different weekly frequencies of concurrent training on muscular power and muscle quality in previously trained elderly men. Additionally, to analyze the training adaptation more comprehensively, we assessed blood pressure and upper-arm vascular function as secondary outcomes. Based on previously studies [14, 15], the operational hypothesis was that concurrent training performed twice a week or three times a week would produce similar muscular adaptations.

## MATERIALS AND METHODS

### Participants

This study enrolled volunteers aged 60 years or older, who have been previously engaged in a regular concurrent training program. The exclusion criteria included any history of neuromuscular, metabolic, hormonal or cardiovascular diseases. Medical evaluations were performed using a health status questionnaire and a maximal exercise test with 12-lead electrocardiography. Participants were advised to maintain their usual dietary intake throughout the study. Eleven patients were taking anti-hypertensive agents during the study, and they were asked to maintain their medication use throughout the study. All participants were informed about the study, including its potential risks and the discomforts related to the procedures, and provided written informed consent. This randomized clinical trial was conducted according to the Declaration of Helsinki and was approved by the local institutional review board (protocol number: 120196).

### Experimental design

In a crossover randomized clinical trial, designed to compare the physiological effects of different concurrent training volumes in elderly men, we assessed a group of trained volunteers. They have previously participated in 12 weeks of periodized resistance and aerobic training three times per week, performing both exercise types in the same training session [18, 19]. After the first 12 weeks, they performed an additional eight weeks of concurrent training three times per week at a constant volume and intensity (25 min of aerobic training at self-selected intensity and two sets of 10-12 repetitions of resistance training using the load correspondent to 15 repetition maximum-RM) to maintain training adaptations.

Therefore, after the 20 weeks of training, participants were eligible to be enrolled in the current trial of 10 weeks of concurrent training. They were randomly assigned to perform aerobic and resistance training either twice a week (2·week^-1^; n=12) or three times per week (3·week^-1^; n=12). To achieve balance across the groups, a computer-based randomization list was generated in permuted blocks of four. The randomized lists were generated by an independent collaborator before the beginning of the trial and were maintained outside the clinic setting. Participants had access to their weekly training frequency only on the first day of experimental sessions.

The aerobic and resistance exercises were performed during the same day/session, starting with resistance exercises and immediately followed by the aerobic exercise. The 2·week^-1^ group trained on Mondays and Fridays, and the 3·week^-1^ group trained on Mondays, Wednesdays, and Fridays. The resistance training was performed using three sets per exercise at intensities between 6-12 RM. The aerobic training lasted 30 min and was performed at 85-95% of heart rate that corresponded to the second ventilatory threshold that was determined by a maximal incremental exercise test on a cycle ergometer.

The concurrent training periodization was used to construct the exercise training programs, as previously described[16]. Despite the differences in the number of training sessions per week, both groups performed with the same intensity and volume (i.e., number of sets, repetitions and time) per session of concurrent training throughout the study. All training sessions were closely supervised by at least 2 experienced personal trainers.

### Measurements

The testing sessions were conducted at the same time, daily. The environmental conditions (e.g., room temperature at 22-24 °C) were kept constant during the tests.

The counter movement jump test was used to evaluate the maximal dynamic power. Using a force platform (OR6-WP, AMTI; Watertown, MA), participants were familiarized to the procedure by performing several jumps to learn the correct technique and to be able to execute three correct, valid jumps. They were instructed to stand with their feet approximately hip-width apart, hands on their hips and to perform the eccentric and concentric phases at maximum speed, flexing the knees at an angle of approximately 90° before starting the concentric phase. Each participant performed a specific warm-up with 3-5 jumps. Three attempts were executed, with 30-60 seconds of rest intervals between the attempts. The greatest jump height was used for the data analyses and was determined by the following equation provided by Asmussen and Bonde-Petersen[20]: jump height=(flight time)2*1.226, using the SAD32 software (Mechanical Measurements Laboratory; UFRGS; Porto Alegre, Brazil). Signal processing included filtering with a fifth-order low-pass Butterworth filter and a cutoff frequency of 30 Hz. Participants were instructed to use the same sport shoes during the pre and post intervention. The test-retest reliability coefficient was 0.82.

The isokinetic concentric knee extension peak torque was measured at speeds of 60°^.^s^-1^ and 180°^.^s^-1^ using a Cybex Norm II Isokinetic Dynamometer (Lumex Co, Ronkonkoma, NY, USA). The participants were positioned seated with their hips and thighs firmly strapped to the seat of the dynamometer, with the hip angle at 85°. After that, a warm-up of 15 submaximal repetitions at 120°^.^s^-1^ was performed. Then, maximal sets of five repetitions at the two speeds were performed with a five min interval between the sets. The contraction with the highest torque value was used in the data analyses. The test-retest reliability coefficients (ICC) were over 0.95 for both velocities.

Muscle quality was evaluated by the quotient between the maximal dynamic strength, which was evaluated through the one-repetition maximal test (1RM) of the knee extensors, and the quadriceps femoris muscle thickness, which was evaluated by ultrasonography (Philips, VMI, Belo Horizonte, MG, Brazil). The 1RM was determined from no more than five attempts with a four-minute recovery between attempts. The muscle thickness was determined by the sum of the muscles vastus lateralis, vastus medialis, vastus intermedius and rectus femoris. A detailed description of the 1RM and muscle thickness testing procedures has been described elsewhere[16].

Brachial blood pressure was measured in triplicate from the dominant arm using a valid calibrated oscillometric automatic device (Dinamap 1846 SX/P; Critikon, FL). Forearm blood flow was measured by venous occlusion plethysmography (D.E. Hokanson, Belleview, WA) in the nondominant forearm. A rapid inflator cuff was used in the upper arm to occlude venous outflow (50-60 mmHg), and three blood flow recordings were performed each minute for 3 min. During the measurements, the blood pressure was measured each minute. After the baseline measurement, reactive hyperemia was evaluated. The blood pressure cuff was inflated to 250 mmHg to institute arterial occlusion for 5 min and was then deflated. Reactive hyperemia was calculated using the peak blood flow after the five-minute occlusion, and the forearm vascular resistance was calculated as the mean blood pressure divided by forearm blood flow[21]. All recordings were manually traced by an investigator, who was blinded to the assigned group. Prior to the study, the reproducibility of the forearm blood flow measurements was determined in a sample of 10 healthy individuals with intraday and interday coefficients of variation of 6.9 and 9.2%, respectively.

### Statistical Analyses

Descriptive data are reported as the mean±SD. A normal distribution and homogeneity parameters were assessed with Shapiro-Wilk and Levene tests. A t-test was used to compare the load lifted during the resistance sessions in each mesocycle. The training-related effects were assessed using a two-way analysis of variance (ANOVA) with repeated measures (group × time). Statistical significance was set at α=0.05, and the SPSS statistical software package (version 17.0) was used to analyze all data.

## RESULTS

One participant dropped out from the 2·week^-1^ group due to personal issues. All the remaining participants completed the protocol and had excellent attendance records (19.7 of 20 sessions (99%) in the 2·week^-1^ group and 29.1 of 30 sessions (97%) in the 3·week^-1^ group). The participant’s characteristics are shown in Table 1. There were no significant differences in the average weight lifted during each of the resistance training sessions between the groups in any of the mesocycles (Table 2).

**Table 1:** Characteristics of the participants [mean ±SD or absolute frequency]

	2·week^-1^ (n=11)		3·week^-1^ (n=12)	

	Pre-training	Post-training	Pre-training	Post-training
Age, y	63.2 ± 2.2	-	65.7 ± 5.7	-
Height, m	1.75 ± 0.6	-	1.69 ± 0.3	-
Body mass, kg	81.4 ± 10.5	80.9 ± 10.2	76.1 ± 6.3	75.9 ± 7.6
Body fat, %	27.8 ± 2.5	25.8 ± 3.7	26.2 ± 2.9	25.2 ± 2.6
VO_2peak_, ml.kg.min^-1^	22.3 ± 4.7	27.2 ± 4.5^*^	25.8 ± 4.9	29.4 ± 5.4^*^
Anti-hypertensive Medications (n)	6	6	5	5

VO2_peak_=peak oxygen consumption; Resistance-aerobic twice a week group (2·week^-1^) and resistance-aerobic three times a week group (3·week^-1^).

* p<0.01 vs Pre.

**Figure 1.**
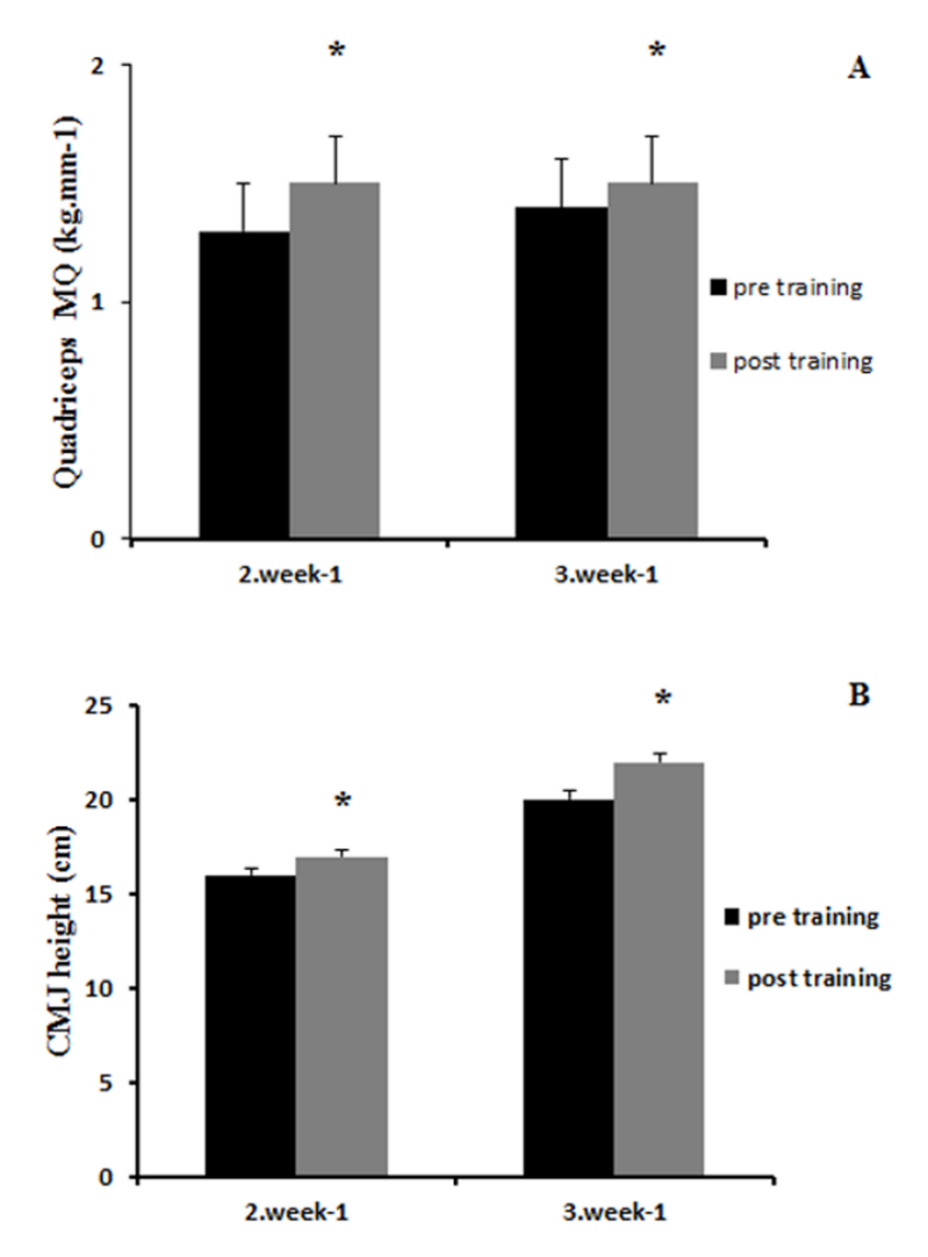
**Changes in Muscle Quality (MQ) (A) and Countermovement jump (CMJ) height (B) with concurrent exercise training**. Resistance-aerobic twice a week group (2·week^-1^) and resistance-aerobic three times a week group (3·week^-1^). Values are means ± SEM. * P<0.05 vs. Pre.

Muscle quality, isokinetic muscular torque, and counter movement jump height were similar at the baseline. Both training groups experienced significant increases in muscle quality and counter movement jump height (Fig. 1). Isokinetic peak torque at slow velocity (60°^.^s^-1^) increased significantly in both groups (2·week^-1^: 193 ± 30 to 201 ± 37 Nm, and 3·week^-1^: 187 ± 30 to 191 ± 19 Nm, *p* = 0.036). Similarly, peak torque at fast velocity (180°^.^s^-1^) increased in both groups after training (2·week^-1^: 125 ± 21 to 134 ± 22 Nm, and 3·week^-1^: 123 ± 24 to 124 ± 18 Nm, *p* = 0.014). No significant differences in the magnitudes of improvements were found between the groups.

**Table 2:** Load lifted per day (kg) during the resistance training sessions in each mesocycle in the periodized resistance training

	Knee extensors			Elbow flexors		

	2/week	3/week	*P* value	2/week	3/week	P value
Mesocycle 1	56.6 ± 12.7	54.1 ± 9.7	0.60	17.1 ± 2.5	16.5 ± 3.0	0.62
Mesocycle 2	65.2 ± 13.2	65.2 ± 10.5	0.99	19.3 ± 2.9	18.1 ± 3.1	0.36
Mesocycle 3	76.2 ± 15.1	75.6 ± 10.6	0.91	22.3 ± 3.2	20.7 ± 3.4	0.27

Data are means ± SD. Resistance-aerobic twice a week group (2·week^-1^) and resistance-aerobic three times a week group (3·week^-1^).

Hemodynamic measures are presented in Table 2. There were no significant baseline differences in any of the cardiovascular measures between the groups. Basal forearm blood flow decreased and vascular resistance increased with concurrent training in both groups, but with no differences between the groups. Brachial blood pressure and reactive hyperemia did not change with concurrent training in either group.

## DISCUSSION

The primary finding of the present study was that the concurrent exercise training performed twice a week produced similar adaptations in muscular power and muscle quality to the concurrent training performed three times per week in previously trained elderly men. This is in spite of the fact that the overall training volume was substantially lower in the twice a week group. In fact, we investigated a group who previously participated in 20 weeks concurrent training three times per week to demonstrate whether these individuals can increase their physical function even with a reduced training frequency. Our present findings may have an important implication for exercise prescription for regularly exercising older adults because relevant increases in muscle power and muscle quality are associated with functional capacity and may be achieved with a lower volume of exercise even in previously trained individuals.

**Table 3:** Forearm hemodynamics and blood pressure with concurrent training.

	2·week^-1^ (n=11)		3·week^-1^ (n=12)	

	Pre-training	Post-training	Pre-training	Post-training
FBF, ml.100ml^-1^.min^-1^	3.2 ± 0.5	2.7 ± 0.8^*^	3.4 ± 0.6	3.0 ± 0.7^*^
FVR, U	29.8 ± 5.1	35.6 ± 7.7^*^	28.9 ± 7.8	33.9 ± 7.3^*^
RH, ml.100ml^-1^.min^-1^	10.6 ± 1.6	9.7 ± 3.2	10.5 ± 2.4	10.7 ± 2.9
Systolic BP, mm Hg	123 ± 18	123 ± 15	132 ± 17	137 ± 14
Diastolic BP, mm Hg	75 ± 9	76 ± 9	73 ± 10	76 ± 9
Mean BP, mm Hg	91 ± 10	92 ± 11	92 ± 11	95 ± 10

Data are means ± SD; FBF, forearm blood flow; FVR, forearm vascular resistance; RH, reactive hyperemia; BP, blood pressure. Resistance-aerobic twice a week group (2·week^-1^) and resistance-aerobic three times a week group (3·week^-1^).

* P<0.01 *vs.* Pre

It has been suggested that muscular power is a more discriminant predictor of functional performance in older adults than muscle strength[6]. The present study demonstrated improvements in both vertical jump and isokinetic peak torque at fast velocity, two important methods used to assess lower extremity muscular power in older individuals. Moreover, 2·week^-1^ and 3·week^-1^ programs produced similar improvements in muscular power, reinforcing the efficiency of lower frequencies of concurrent training in the elderly[16]. Considering that the capacity to perform daily activities, such as walking, climbing stars, and gardening among others, is critical for maintaining independent functioning in elderly individuals[6], the present results highlight the efficacy of concurrent exercise training to enhancing muscular power in previously trained elderly men.

Aging is associated with declines in the force per unit of muscle mass (i.e., muscle quality)[7, 22], and declines in muscle quality are associated with a reduced functional capacity in elderly populations[7, 22, 23]. A recently published study showed associations between muscle quality and functional tasks, such as 30-s sit-to-stand, and 8 foot up-and-go after a short-term resistance training in untrained elderly[23]. Our results showed that combined resistance and aerobic exercises significantly improved muscle quality and that training twice a week or three times a week elicited similar magnitudes of improvements in muscle quality. To the best of our knowledge, this is the first study to investigate the concurrent training effects on muscle quality in previously trained elderly men.

A recent meta-analysis showed that concurrent training is effective in reducing blood pressure, and the hypotensive effects of exercise were observed after exercise programs with shorter durations[13]. The lack of change in blood pressure and hemodynamic measures observed in the present study may have been due to the previous training status of participants. Participants were previously trained in both aerobic and resistance training, so it is possible that hemodynamic improvements had already been achieved, as hemodynamic changes resulting from exercise training are fairly quick and robust in the first few months of training in previously untrained individuals[24].

A previous cross-sectional study showed that resistance-trained older individuals demonstrate greater muscle mass and higher basal leg blood flow compared with age-matched sedentary adults[25, 26]. One unexpected finding in the present study was that both groups experienced significant reductions in basal forearm blood flow with the concurrent training in both groups, especially considering that lean body mass, i.e., metabolically active tissue, increased with the concurrent training. However, changes with exercise training were relatively small in magnitude and may be within the measurement error of the plethysmographically measured forearm blood flow.

The present results bring important implications for exercise prescription. First, the combination of resistance and aerobic exercises is beneficial for developing and maintaining neuromuscular and cardiovascular functions in the previously trained elderly. Second, these functional benefits can be achieved even if the concurrent exercise was performed only twice a week in individuals who previously trained three times per week. Finally, the use of the current exercise program can help in counteracting the marked reductions in the cardiovascular and neuromuscular system that occur with advancing age and improve mobility and function in elderly populations. However, some limitations should be taken into account when interpreting the present results. First, a non-exercising control group was not included in the study. Second, our sample consisted of men only, therefore limiting the generalization of our findings to the female population. Third, a more comprehensive comparison would have been possible with the inclusion of a once weekly training group (i.e., weekend warriors), which could indicate whether a very low weekly training volume induces improvements in previously trained older individuals.

### Conclusion

In previously trained older men, concurrent training performed twice a week produces similar responses in muscle power and muscle quality when compared with the same program performed three times per week in elderly men. Our results may have important practical applications in exercise prescription because lower frequencies of exercise associated with less time dedicated to exercise may facilitate a better adherence and compliance in this high-risk population.

## References

[1] Izquierdo M, Hakkinen K, Anton A, Garrues M, Ibanez J, Ruesta M, et al (2001). Maximal strength and power, endurance performance, and serum hormones in middle-aged and elderly men. Medicine and science in sports and exercise, 33: 1577-1587 11528348 10.1097/00005768-200109000-00022

[2] Lauretani F, Russo CR, Bandinelli S, Bartali B, Cavazzini C, Di Iorio A, et al (2003). Age-associated changes in skeletal muscles and their effect on mobility: an operational diagnosis of sarcopenia. Journal of applied physiology, 95: 1851-1860 14555665 10.1152/japplphysiol.00246.2003

[3] Lee DC, Sui X, Artero EG, Lee IM, Church TS, McAuley PA, et al (2011). Long-term effects of changes in cardiorespiratory fitness and body mass index on all-cause and cardiovascular disease mortality in men: the Aerobics Center Longitudinal Study. Circulation, 124: 2483-2490 22144631 10.1161/CIRCULATIONAHA.111.038422 PMC3238382

[4] Aagaard P, Magnusson PS, Larsson B, Kjaer M, Krustrup P (2007). Mechanical muscle function, morphology, and fiber type in lifelong trained elderly. Medicine and science in sports and exercise, 39: 1989-1996 17986907 10.1249/mss.0b013e31814fb402

[5] Steib S, Schoene D, Pfeifer K (2010). Dose-response relationship of resistance training in older adults: a meta-analysis. Medicine and science in sports and exercise, 42: 902-914 19996996 10.1249/MSS.0b013e3181c34465

[6] Reid KF, Fielding RA (2012). Skeletal muscle power: a critical determinant of physical functioning in older adults. Exercise and sport sciences reviews, 40: 4-12 22016147 10.1097/JES.0b013e31823b5f13 PMC3245773

[7] Misic MM, Rosengren KS, Woods JA, Evans EM (2007). Muscle quality, aerobic fitness and fat mass predict lower-extremity physical function in community-dwelling older adults. Gerontology, 53: 260-266 17446711 10.1159/000101826

[8] Foldvari M, Clark M, Laviolette LC, Bernstein MA, Kaliton D, Castaneda C, et al (2000). Association of muscle power with functional status in community-dwelling elderly women. The journals of gerontology. Series A, Biological sciences and medical sciences, 55: M192-199 10.1093/gerona/55.4.m192 10811148

[9] Cadore EL, Pinto RS, Bottaro M, Izquierdo M (2014). Strength and endurance training prescription in healthy and frail elderly. Aging and disease, 5: 183-195 24900941 10.14336/AD.2014.0500183 PMC4037310

[10] Santos-Parker JR, LaRocca TJ, Seals DR (2014). Aerobic exercise and other healthy lifestyle factors that influence vascular aging. Advances in physiology education, 38: 296-307 25434012 10.1152/advan.00088.2014 PMC4315444

[11] Seals DR, Desouza CA, Donato AJ, Tanaka H (2008). Habitual exercise and arterial aging. Journal of applied physiology, 105: 1323-1332 18583377 10.1152/japplphysiol.90553.2008 PMC2576026

[12] Picon RV, Fuchs FD, Moreira LB, Fuchs SC (2013). Prevalence of hypertension among elderly persons in urban Brazil: a systematic review with meta-analysis. American journal of hypertension, 26: 541-548 23467209 10.1093/ajh/hps076

[13] Cornelissen VA, Smart NA (2013). Exercise training for blood pressure: a systematic review and meta-analysis. Journal of the American Heart Association, 2: e004473 23525435 10.1161/JAHA.112.004473 PMC3603230

[14] Izquierdo M, Ibanez J, K HA, Kraemer WJ, Larrion JL, Gorostiaga EM (2004). Once weekly combined resistance and cardiovascular training in healthy older men. Medicine and science in sports and exercise, 36: 435-443 15076785 10.1249/01.mss.0000117897.55226.9a

[15] Fisher G, McCarthy JP, Zuckerman PA, Bryan DR, Bickel CS, Hunter GR (2013). Frequency of combined resistance and aerobic training in older women. Journal of strength and conditioning research / National Strength & Conditioning Association, 27: 1868-1876 10.1519/JSC.0b013e31827367e0 PMC4066209 22996024

[16] Ferrari R, Kruel LF, Cadore EL, Alberton CL, Izquierdo M, Conceicao M, et al (2013). Efficiency of twice weekly concurrent training in trained elderly men. Experimental gerontology, 48: 1236-1242 23933066 10.1016/j.exger.2013.07.016

[17] Mazzeo RS, Tanaka H (2001). Exercise prescription for the elderly: current recommendations. Sports medicine, 31: 809-818 11583105 10.2165/00007256-200131110-00003

[18] Cadore EL, Izquierdo M, Alberton CL, Pinto RS, Conceicao M, Cunha G, et al (2012). Strength prior to endurance intra-session exercise sequence optimizes neuromuscular and cardiovascular gains in elderly men. Experimental gerontology, 47: 164-169 22178632 10.1016/j.exger.2011.11.013

[19] Cadore EL, Izquierdo M, Pinto SS, Alberton CL, Pinto RS, Baroni BM, et al (2013). Neuromuscular adaptations to concurrent training in the elderly: effects of intrasession exercise sequence. Age, 35: 891-903 22453934 10.1007/s11357-012-9405-y PMC3636398

[20] Asmussen E, Bonde-Petersen F (1974). Storage of elastic energy in skeletal muscles in man. Acta physiologica Scandinavica, 91: 385-392 4846332 10.1111/j.1748-1716.1974.tb05693.x

[21] Umpierre D, Stein R, Vieira PJ, Ribeiro JP (2009). Blunted vascular responses but preserved endothelial vasodilation after submaximal exercise in chronic heart failure. European journal of cardiovascular prevention and rehabilitation : official journal of the European Society of Cardiology, Working Groups on Epidemiology & Prevention and Cardiac Rehabilitation and Exercise Physiology, 16: 53-59 10.1097/HJR.0b013e32831c8489 19188808

[22] Granacher U, Gruber M, Gollhofer A (2010). Force production capacity and functional reflex activity in young and elderly men. Aging clinical and experimental research, 22: 374-382 19966537 10.1007/BF03337733

[23] Pinto RS, Correa CS, Radaelli R, Cadore EL, Brown LE, Bottaro M (2014). Short-term strength training improves muscle quality and functional capacity of elderly women. Age, 36: 365-372 23881608 10.1007/s11357-013-9567-2 PMC3889909

[24] DeSouza CA, Shapiro LF, Clevenger CM, Dinenno FA, Monahan KD, Tanaka H, et al (2000). Regular aerobic exercise prevents and restores age-related declines in endothelium-dependent vasodilation in healthy men. Circulation, 102: 1351-1357 10993851 10.1161/01.cir.102.12.1351

[25] Anton MM, Cortez-Cooper MY, DeVan AE, Neidre DB, Cook JN, Tanaka H (2006). Resistance training increases basal limb blood flow and vascular conductance in aging humans. Journal of applied physiology, 101: 1351-1355 16840576 10.1152/japplphysiol.00497.2006

[26] Miyachi M, Tanaka H, Kawano H, Okajima M, Tabata I (2005). Lack of age-related decreases in basal whole leg blood flow in resistance-trained men. Journal of applied physiology, 99: 1384-1390 15961613 10.1152/japplphysiol.00061.2005

